# The Role of Microglia and the Nlrp3 Inflammasome in Alzheimer's Disease

**DOI:** 10.3389/fneur.2020.570711

**Published:** 2020-09-18

**Authors:** Kendra L. Hanslik, Tyler K. Ulland

**Affiliations:** ^1^Neuroscience Training Program, University of Wisconsin, Madison, WI, United States; ^2^Department of Pathology and Laboratory Medicine, University of Wisconsin, Madison, WI, United States

**Keywords:** Alzheimer's disease, microglia, Nlrp3 inflammasome, neuroinflammation, neurodegeneration

## Abstract

Alzheimer's disease (AD) is the most prevalent form of late-onset dementia. AD affects the health of millions of people in the United States and worldwide. Currently, there are no approved therapies that can halt or reverse the clinical progression of AD. Traditionally, AD is characterized first by the appearance of amyloid-β (Aβ) plaques followed by the formation of intraneuronal neurofibrillary tangles (NFTs) composed of hyperphosphorylated tau (p-tau). These lesions are linked to synapse loss and eventual cognitive impairment. Additionally, microgliosis is consistently found in regions of the brain with AD pathology. The role of microglia in AD onset and progression remains unclear. Several recent reports indicate that the assembly of the multi-protein complex known as the NOD, LRR, and pyrin-domain containing 3 (Nlrp3) inflammasome by microglia results in apoptosis spec-like protein containing a CARD (Asc) spec formation, which then nucleates new Aβ plaques, thus amplifying Aβ-associated pathology. NFTs can also activate the Nlrp3 inflammasome leading to enhanced tau-associated pathology. Here, we will review the role of microglia and the activation of the inflammasome in the innate immune response to AD.

## Introduction

**Figure 1:**
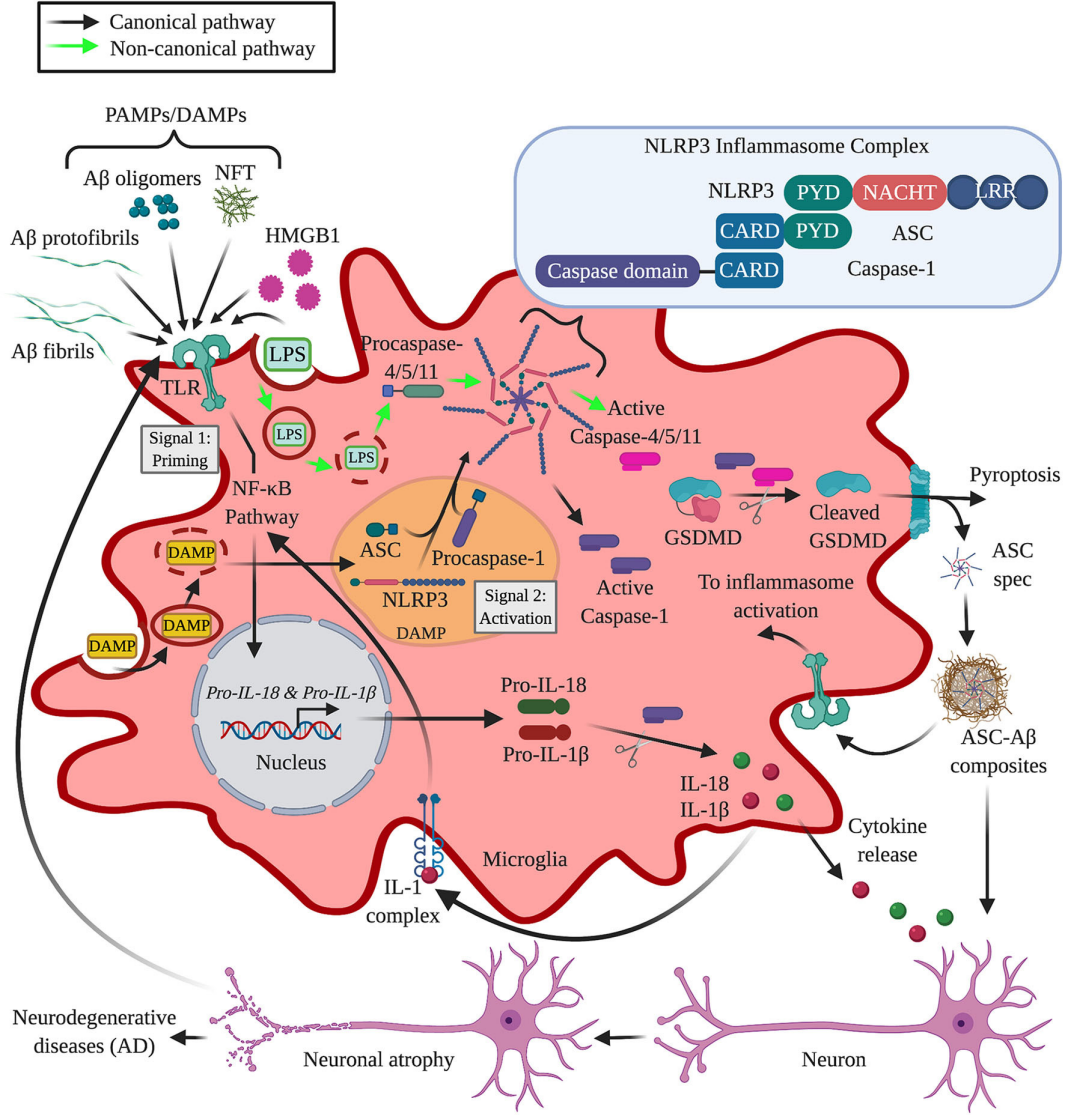
Role of microglia and canonical NLRP3 inflammasome in Alzheimer's disease. Cytokines, pathogen-associated molecular patterns (PAMPs), or danger-associated molecular patterns (DAMPs) bind cell surface receptors [e.g., toll-like receptors (TLRs)] on the microglia, leading to activation of the nuclear factor-κB (NF-κB) pathway (signal 1). The activation of the NF-κB pathway promotes a signaling cascade, resulting in the expression of cytokines such as pro-IL-1β and pro-IL-18 as well as NLR family pyrin domain containing 3 protein (Nlrp3). Through the canonical activation pathway, Nlrp3 oligomerizes in response to an internalized, cytosolic danger signal (DAMP) (signal 2) then recruits Asc and procaspase-1, resulting in inflammasome assembly (Asc-spec assembly) and caspase-1 autoactivation. Activated caspase-1 then cleaves pro-IL-1β or pro-IL-18 and gasdermin (GSDMD). GSDMD cleavage is also induced by caspases 4/5/11 through a non-canonical activation pathway that detects internalized, cytosolic LPS, and other PAMPs/DAMPs that bypass membrane-bound pattern recognition receptors such as TLRs. Subsequently, GSDMD induces pyroptosis, presumably releasing Asc-specs that cross-seed extracellular amyloid beta (Aβ) plaques and creates Asc-Aβ composites that induce a feed-forward cycle amplifying the proinflammatory response. In turn, proinflammatory cytokines including IL-1β and IL-18 induce neuronal damage and death, causing neurodegeneration. Degraded neurons can then trigger a feedback loop by activating microglia. IL-1R complexes are also activated, creating a positive feedback loop that drives additional pro-IL-1β production and primes local microglia for inflammasome activation. AD, Alzheimer's disease; IL, interleukin; NFT, neurofibrillary tangles; ASC, apoptosis-associated spec-like protein containing a CARD; CARD, caspase-activation and recruitment domain; LRR, leucine-rich repeat; NACHT, nucleotide-binding oligomerization domain; PYD, Pyrin domain; HMGB1, high mobility group box 1; LPS, lipopolysaccharide; NF-κB, nuclear factor kappa light chain enhancer of activated B cells created with BioRender.

Alzheimer's disease is the most common form of dementia. AD results in neuronal death likely caused by an accumulation of senile plaques primarily composed of amyloid-β (Aβ) peptides, first observed by Alois Alzheimer (1). Plaques promote an environment conducive to forming intraneuronal tau aggregates known as neuritic plaque tau, (NP) tau, and in more advanced stages of AD, neurofibrillary tangles (NFTs) (2, 3). Recent evidence suggests that neuroinflammation, mediated through increased levels of pro-inflammatory products released from innate immune cells, e.g., microglia, contribute to AD, and precedes Aβ plaque deposition and AD onset (4, 5). Microglial dysfunction caused by prolonged amyloid-induced microglial activation may also contribute to AD (6). Microglia are also crucial for maintenance and upholding homeostasis within the brain (7). Upon activation in numerous pathological conditions, including AD, microglial function, and morphology change dramatically (7). Monomeric and oligomeric forms of Aβ as well as tau aggregates such as NFTs activate microglia in AD (Figure 1). Additionally, activation of pattern recognition receptors (PRRs) expressed by microglia can influence AD pathology (8). Microglia and other innate immune cells express several toll-like receptors (TLRs), which when activated, subsequently result in the activation of NF-κB and the production of pro-inflammatory cytokines (9). Additionally, microglia also express several intracellular PRRs that are not membrane-bound such as nucleotide-binding domain and leucine-rich repeat-containing receptors (NLRs) family of receptors, absent in melanoma 2 (AIM2)-like receptors (ALRs) family of receptors and the tripartite motif-containing (TRIM) family member pyrin are known to initiate the assembly of the multi-protein complex inflammasome (10, 11). In most cases the inflammasome complex contains apoptosis spec like protein containing a caspase recruitment domain (Asc), and is also known as an Asc spec (12).

In this review, we will primarily discuss the role of inflammasome activation by microglia in Alzheimer's disease with a special focus on Nlrp3 inflammasome activation.

## Innate Immune Response in AD

Microglia originate from primitive macrophages that exit the yolk sac and colonize the neuroepithelium and are the primary immunocompetent cells found within the brain (13, 14). In a normal physiological state, microglia play a critical role in multiple developmental events within the central nervous system (CNS) such as the establishment of neural circuits, synaptic pruning and remodeling, and neurogenesis (15–21). Microglia are also responsible for clearing cellular debris and aggregate-prone proteins including Aβ as well as harmful bacteria and viruses through phagocytosis in diseased states (22). Microglia within a normal adult mouse brain are highly dense in gray matter areas including the hippocampus, basal ganglia, substantia nigra, and olfactory cortex (23). Throughout these areas, the number and role of microglia is highly regulated by the local microenvironment and their interactions with surrounding cells such as neurons, astrocytes, and oligodendrocytes (24).

Studying key Alzheimer's risk genes has provided critical insights into the function of microglia and how microglia modulate pathology in AD. It is known that several genes, expressed exclusively by microglia in the brain, such as CD33, a sialic-acid-binding immunoglobulin-like lectin (SIGLECs), and triggering receptor expressed on myeloid cells 2 (Trem2) carry single nucleotide polymorphisms (SNPs) that influence the risk for developing AD (7). CD33, a PRR on the cell's surface that recognizes sialylated glycoproteins and gangliosides, promotes Aβ deposition and plaque formation (25). Griciuc et al. (25) also found that CD33 expression impedes microglia's uptake and clearance of amyloid-β 42 (Aβ42), resulting in a larger plaque burden. Inhibition of CD33 using a subtype-selective sialic acid mimetic mitigates Aβ plaque-associated pathology by increasing Aβ plaque phagocytosis (26). Trem2 is another microglial surface receptor, it binds phospholipids and other polyanionic ligands and detects changes in the lipid microenvironment (27). Studies have shown that Trem2-deficient AD mouse models exhibit decreased clustering of microglia around plaques and increased neuritic damage, suggesting that this gene is crucial for containing early plaque diffusion (28, 29). Conversely, Trem2 expression in response to tau has been shown to enhance AD-like pathology (30, 31). So although it appears that Trem2 is important in enhancing microglial responses during AD-associated pathology it remains controversial whether Trem2 expression is overall beneficial or pathogenic in AD (29, 31–34). It is also important to note that many of these findings still require replication in patient samples to confirm the roles of these molecules in AD pathogenesis.

Microglia form a lattice throughout the brain and express an array of PRRs, which sense changes in the brain's environment through the detection of danger-associated molecular patterns (DAMPs) and pathogen-associated molecular patterns (PAMPs) among other stimuli (10). When a shift in the microenvironment is detected, these receptors send converging signals that promote a spectrum of microglial responses from surveillance to activation (35). As microglia survey their environment they rapidly extend and retract their filopodia-like processes within an area of the parenchyma, allowing them to survey that microenvironment (35). When activated, microglia adopt different morphologies, and produce various cytotoxic molecules including pro-inflammatory cytokines and inflammatory mediators consisting of nitric oxide (NO) and reactive oxygen species (ROS) (22, 36, 37). In summary, elucidating the links between innate immune activation and microglia's inflammatory responses concomitant with inflammasome activation, is becoming a crucial research area to better understand AD pathology and to find new therapeutic targets that could impede or slow its progression.

## Nlrp3 Inflammasome Activation

Several inflammasomes have been implicated in neurodegenerative diseases, the NOD-, LRR-, and pyrin domain-containing 3 (NLRP3) inflammasome in particular has been shown to play a key role in the development and progression of Aβ-plaque formation as well as tau-induced pathology, which has been demonstrated in both post-mortem AD patient brain tissue and *in vivo*/*in vitro* transgenic mouse studies (37–42). The inflammasome is a multimeric protein complex that is most commonly composed of a sensor, an adaptor, and the downstream effector caspase-1 (12). Each inflammasome is named according to the sensor molecule that initiates activation and is activated through two signals that first prime and then activate the complex (43) (Figure 1). Upon activation, Nlrp3, and the majority of other structurally related receptors such as other NLRs, AIM2, or pyrin can form homotypic PYD-PYD or CARD-CARD interactions with the adaptor Asc (apoptosis-associated spec-like protein containing a caspase activating and recruiting domain) (44, 45). The interactions between these molecules are composed of an N-terminal pyrin domain (PYD) and C-terminal caspase-activation and recruitment domain (CARD) and result in the formation of a ring-like perinuclear complex called an Asc “spec,” a typical indicator of canonical inflammasome activation (12, 46). Following inflammasome activation, Asc recruits procaspase-1 through interactions with the CARD domain of caspase-1 (47). Procaspase-1 is then converted into its bioactive form through proximity-induced autocatalysis, producing mature caspase-1 that cleaves pro-IL-1β and pro-IL-18 into their respective secreted forms (10, 48). Caspase-1 also triggers the cleavage of pore-forming Gasdermin D (GSDMD), which induces a lytic, pro-inflammatory form of cell death called pyroptosis (49, 50). Generally, priming and activation of the inflammasome occurs in response to two different signals, however, it is possible that one molecule can deliver both signals. For example, LPS can initiate both the formation of the canonical and non-canonical Nlrp3 inflammasome involving human caspases 4/5 and mouse caspase-11 rather than caspase-1 (51–53). Non-canonical Nlrp3 inflammasome activation serves as another layer of defense for pathogens that have evolved to bypass membrane-bound PRRs such as TLR4 (54) (Figure 1). This form of activation is prompted by caspases' 4/5/11 detection of cytosolic lipopolysaccharide (LPS), which induces pyroptotic cell death through GSDMD cleavage (43). In AD, the Nlrp3 inflammasome is responsible for the maturation of caspase-1, which is in turn responsible for the maturation and secretion of pro-inflammatory cytokines such as IL-1β and IL-18 that can activate signaling pathways resulting in neuroinflammation and neuronal death (5, 12).

## Nlrp3 Inflammasome In Normal Aging

The concept of “inflamm-aging,” the low-grade chronic inflammatory state that accompanies aging, is a recent topic of interest (55). During aging, inflammasome activation can be triggered by local microenvironment changes associated with aging microglia (56). For example, aging microglia exhibit altered cytokine production, making microglial cells more susceptible to adopting a pro-inflammatory state that also primes the cells for inflammasome activation (56, 57). Aging microglial cells also have an increased accumulation of lipofuschin that has been associated with increased oxidative stress, which may cause microglia to lose their neuroprotective potential and contribute to age-related pathology (58, 59). Additional evidence suggests that components of the inflammasome including caspase-1, caspase-11, Asc, and IL-1β are increased in the cytosolic fraction of hippocampal lysates in aged mice, suggesting that inflammasome formation contributes to inflammation in aging (60). Consistent with the inflamm-aging hypothesis, Youm et al. found that reducing the Nlrp3 inflammasome-dependent pro-inflammatory cascade alleviated age-associated degenerative changes across multiple organs (61). This study also showed that *Nlrp3* gene expression was lower in younger microglia compared to their senile counterparts (61). Thus, the status of inflamm-aging in the brain may be associated with changes in aging microglia prompted by local microenvironment and systemic environment states that induce inflammasome activation.

## Nlrp3 Inflammasome and the Microbiome

Emerging evidence has highlighted cross-talk interactions between the gut microbiome and the brain (62, 63). In AD, the composition of gut microbiota can influence the development of or exacerbate the pathology associated with AD (64, 65). The role inflammasomes play within the gut-brain crosstalk is less clear. A newly published study that transplanted gut microbiota from AD patients to either APP/PS1 mice, a double transgenic mouse that carries chimeric mouse/human amyloid precursor protein (APP) and human presenilin 1 (PS1) mutations associated with familial AD, or wild type mice, demonstrated that the transplantation of the gut microbiome of an AD patient can influence AD pathology and Nlrp3 inflammasome activation. This study found that APP/PS1 mice receiving a transplant of the gut microbiome from an AD patient had increased expression of Nlrp3 in their intestinal tract and increased levels of inflammatory factors such as IL-1β and IL-6 in their peripheral blood (62). These mice also exhibited more severe cognitive impairment compared to those that did not receive the transplant. When the gut microbiome from an AD patient was transplanted into wild type mice, the intestinal expression of Nlrp3 was also upregulated but their cognitive abilities were not significantly altered (62). The microglia in the hippocampi of these mice, however, were still activated and there was still an up-regulated expression of inflammatory factors. Taken together, these studies indicate that gut microbiota modulate inflammatory responses through Nlrp3 inflammasome signaling.

## Nlrp3 Inflammasome in AD

Both the accumulation and deposition of Aβ as well as NFT formation are detected by cytosolic PRRs, prompting Nlrp3 inflammasome activation in microglia (37, 38). The association between microglial Nlrp3 inflammasome activation and fibrillar Aβ was first demonstrated *in vitro* by Halle et al. (66). Their study found that exposing Aβ fibrils to primary mouse microglia induces IL-1β secretion in an Nlrp3-specific manner (66). Recently, soluble Aβ oligomers and protofibrils have also been shown to induce Asc spec formation in primary microglia cells collected from wild type mice (67). Nlrp3 activation has also been linked with tau aggregates in PS19 mice, a mouse model that overexpresses the human tau protein carrying the P301S mutation (39). The association between Nlrp3 inflammasome activation and tau exacerbates and drives tau pathology in AD mouse models (38, 39). Related work by Ising and colleagues was the first to suggest that the Nlrp3 inflammasome forms a link between Aβ plaques and NFTs (38). They showed that Tau22 mice receiving an intracerebral injection of fibrillar Aβ-containing APP/PS1 brain homogenates exhibited increased levels of tau hyperphosphorylation, cleaved caspase-1, IL-1β, and Asc in cerebral samples as well as significantly higher levels of extracellular Asc specs, which have been shown to seed Aβ plaques (37, 38). However, when they injected the same homogenate in Tau22/*Asc*^−/−^ or Tau22/*Nlrp3*^−/−^ mice, tau hyperphosphorylation did not occur and there were lower levels of cleaved caspase-1, IL-1β, and reduced Asc spec formation and release, further verifying that Nlrp3 activation is essential in the Aβ-tau cascade (38). These findings demonstrate a link between both tau and Aβ pathology and confirm Nlrp3 inflammasome activation in Tau22 mice. Additionally, Heneka *et al*. used *Nlrp3*-deficient and *caspase-1*-deficient APP/PS1 mice to show that mice unable to activate the inflammasome were spared from memory deficits and LTP suppression unlike the APP/PS1 mice that exhibited severe deficits in spatial memory formation (68). Their findings showed that Nlrp3 inflammasome activation restricts beneficial microglial clearance functions while *Nlrp3-* or *caspase-1*-deficiency increases microglial plaque phagocytosis (68). Interestingly, new findings support the notion that caspase-1 activation and pro-inflammatory cytokine secretion precede AD pathology, implying that Nlrp3 inflammasome activation is an early pathogenic event in AD (4, 69). Overall, these data indicate that targeting the Nlrp3 inflammasome in human clinical trials are warranted to determine whether inhibition of the inflammasome will hinder Aβ deposition and NFT formation and correlate positively with cognitive outcome measures.

## Nlrp3 Inhibition As a Therapeutic Intervention for AD

As Nlrp3 inflammasome activation through both canonical and non-canonical pathways has been shown to play an important role in the pathology of AD, the Nlrp3 inflammasome has emerged as a possible target for future pharmacological therapies (67, 70, 71). Since Nlrp3 inflammasome activation is a multi-step process, inhibiting Nlrp3 inflammasome activation can be accomplished through several different means including: suppressing molecules that promote inflammasome activation or formation, silencing upstream signals, or by directly or indirectly inhibiting the inflammasome complex formation depending on the molecule targeted (10, 72). Some of the direct inhibitors, which specifically target Nlrp3-Nlrp3, Nlrp3-Asc, or NEK7-Nlrp3 interactions, are ginsenoside Rg3, oridonin, and tranilast (73–75). Ginsenoside Rg3, isolated from *Panax ginseng*, specifically blocks IL-1β secretion and caspase-1 activation by inhibiting LPS priming and Nlrp3 inflammasome assembly (73). In contrast, oridonin, derived from *Rabdosia rubescens*, blocks inflammasome assembly and activation by hindering the NEK7-Nlrp3 interaction, which is crucial for Nlrp3 oligomerization and Asc recruitment to Nlrp3 (74, 76). Tranilast, a historical anti-allergic drug used in the clinic, directly suppresses Nlrp3 inflammasome assembly by blocking Nlrp3 oligomerization (75). Examples of indirect inhibitors include β-hydroxybutyrate (BHB), MCC950, glyburide, and 16673-43-0, a glyburide analog that has no effect on insulin (77–80). BHB hinders inflammasome formation by inhibiting K^+^ efflux, which causes mitochondrial damage and exposure to a mitochondrial-specific phospholipid, cardiolipin, that leads to Nlrp3 activation (77, 81). MCC950, on the other hand, selectively blocks both the canonical and non-canonical Nlrp3 inflammasome activation pathways by impeding the ATP hydrolysis motif (80). Glyburide works by suppressing ATP-sensitive K^+^ channels and caspase-1 activation while its analog, 16673-43-0, achieves inhibition by inducing conformational changes in the inflammasome following Asc's activation or aggregation (79, 82).

Additional methods of inhibiting Nlrp3 inflammasome activation using autophagy-inducing treatments and microRNAs have recently become possible. Some studies have shown that autophagic proteins such as autophagy-related protein 7 (ATG7), microtubule-associated protein 1 light chain 3B (LC3B), and beclin-1 regulates Nlrp3 inflammasome activation by sustaining mitochondrial integrity (83, 84). This data demonstrated that deficiencies in autophagic proteins such as LC3B increases caspase-1 cleavage, Asc spec formation, and IL-1β release in macrophages (83, 84). Thus, by administering autophagy-inducing agents including resveratrol and cannabinoid receptor 2 (CB2R) agonists such as HU-308, Nlrp3 inflammasome activation can be inhibited (85, 86). MicroRNA based post-transcriptional Nlrp3 regulation also prevents inflammasome formation by reducing endogenous Nlrp3 protein levels (87, 88). MicroRNAs are small, conserved single stranded noncoding RNAs that post-transcriptionally regulate gene expression. They bind untranslated regions (UTRs) of transcripts and modify the stability and translation of the target mRNA, producing an inhibitory effect (89). One such example is miR-223, which targets a binding site in the Nlrp3 3′-UTR and was validated *in vitro* in macrophages (87). This study found that miR-223 overexpression inhibits Nlrp3 protein accumulation and IL-1β production from the inflammasome (87). Although each inhibitor has its own mechanism, they all have a similar effect resulting in decreased inflammasome formation, cytokine release, and systemic inflammation.

Within the context of Alzheimer's disease, some of these inhibitors have already been shown to reduce AD-associated pathology (Table 1). For instance, JC-124 and oridonin, two direct Nlrp3 inflammasome inhibitors, have been shown to reduce amyloid burden and microglial activation in AD mouse models (71, 91). Oridonin treatment also showed beneficial effects in attenuating disease pathology by decreasing inflammatory cytokine release in the hippocampus (91). Another study that elevated plasma ketone body levels through an oral dose of medium-chain triglycerides to individuals with AD or mild cognitive impairment reported a significant increase of BHB in serum levels and some subjects exhibited cognitive improvement (93).

**Table 1:** Potential inhibitors of the Nlrp3 inflammasome.

**Type**	**Nlrp3-targeting Agent**	**Tested in AD**	**Neuroprotection Observed**	**Sources**
Direct Inhibitors	Ginsenoside Rg3	N	–^a^	(73, 90)
	JC-124	Y	Y	(71)
	Oridonin	Y	Y	(91)
	Tranilast	N	–	(75)
Indirect Inhibitors	Ketone bodies (i.e., BHB)	Y	Y	(92)
	MCC950	N	–	(80)
	Glyburide	N	–	(82)
	16673-43-0	N	–	(79)
Autophagy-inducing	Resveratrol	N	–	(86)
	HU-308	N	–	(85)
MicroRNAs	miR-223	N	–	(87)
	miR-9	N	–	(88)

a *Neuroprotection observations were not applicable in these studies*.

While these findings seem promising, there are some limitations to consider when employing these agents as a potential therapeutic. For example, unintended immunosuppressive effects may result from the use of inhibitors that target IL-1β secretion or signaling (94, 95). Likewise, inhibitors focused on reducing cytokine secretion alone does not address Nlrp3-induced pyroptosis, which may contribute to additional pathology (72). Finally, compounds that inhibit cytokine secretion will not necessarily mitigate pathology driven by Asc spec formation. Continued Asc spec formation will result in further seeding of extracellular Aβ plaques, amplifying amyloid pathology, and the pro-inflammatory response (37). Overall, these studies suggest that hindering inflammasome assembly is a potential intervention method for attenuating AD pathology with the caveat that inhibition of the inflammasome can have severe unintended results.

## Concluding Remarks

Traditionally, the cognitive decline associated with AD was attributed to the accumulation of amyloid and tau but emerging evidence suggests that neuroinflammation driven by and triggering additional Nlrp3 inflammasome activation is another critical contributor to AD pathology. Additional investigations are needed to gain insight into the contribution of other inflammasome pathways in neurodegeneration. Additional research is also needed to further clarify the complexity of microglia's signaling cascades and to determine how to modify the microglial response as a potential method for managing or treating AD.

While regulation of Nlrp3 inflammasome assembly and activation may be a potential therapeutic approach, it is currently unknown whether targeting inflammasome activation in AD will result in a beneficial or detrimental effect on clinical outcomes such as cognitive measures. It is also important to consider other therapeutic approaches aimed at modifying microglial function. It is possible that the pro-inflammatory response reproduced in many experimental studies and human genetic or RNA sequencing studies may be the product of a secondary pro-inflammatory response from tissue injury induced by neuronal death, the spreading of AD pathology, or glial dysfunction. Considering that there is microglial dysfunction in AD such as metabolic defects as well as decreased microglial motility and chemotaxis, promoting normal microglial function may prove an alternative therapeutic approach (6, 96, 97). Additional approaches may also include inhibiting microglial proteolytic cleavage of P2RY12, a G-coupled protein receptor located on glial cells, whose inhibition has been associated with neuroprotective effects and promoting other anti-inflammatory cytokines such as IL-4 or progranulin, which still remain to be tested and compared to inflammasome inhibitors (98–100).

## Author Contributions

KH conceived the review. KH and TU discussed and contributed to the writing of this review. All authors contributed to the article and approved the submitted version.

## Conflict of Interest

The authors declare that the research was conducted in the absence of any commercial or financial relationships that could be construed as a potential conflict of interest.
